# Aerial surveys of waterbirds in Australia

**DOI:** 10.1038/s41597-020-0512-9

**Published:** 2020-06-10

**Authors:** Richard T. Kingsford, John L. Porter, Kate J. Brandis, Sharon Ryall

**Affiliations:** 1 Centre for Ecosystem Science, School of Biological, Environmental and Earth Sciences, UNSW, Sydney, NSW 2052 Australia; 2 New South Wales Department of Planning, Industry and Environment, 4 Parramatta Square, 12 Darcy Street, Parramatta, 2150 NSW Australia

**Keywords:** Biodiversity, Environmental impact

## Abstract

Tracking long-term environmental change is important, particularly for freshwater ecosystems, often with high rates of decline. Waterbirds are key indicators of freshwater ecosystem change, with groups reflecting food availability (e.g. piscivores and fish). We store waterbird (species abundance, numbers of nests and broods) and wetland area data from aerial surveys of waterbirds across Australia, mostly at the species’ level (∼100 species) from three aerial survey programs: Eastern Australian Waterbird Survey, National Survey and Murray-Darling Basin wetlands. Across eastern Australia, we survey up to 2,000 wetlands annually (October, since 1983), along 10 survey bands (30 km wide), east to west across about one third of Australia. In 2008, we surveyed 4,858 wetlands across Australia and each year (since 2010) we survey the major wetlands in the Murray-Darling Basin. These data inform regulation of hunting seasons in Victoria and South Australia, Game bird culling in NSW, State of the Environment Reporting, environmental assessments, river and wetland management, the status of individual species and identification of high conservation sites.

## Abstract (summary)

**Table:** 

Measurement(s)	population • numbers of waterbird species • brood size • nest count
Technology Type(s)	visual observation method
Factor Type(s)	year • geographic location
Sample Characteristic - Organism	Aves
Sample Characteristic - Environment	wetland area
Sample Characteristic - Location	Australia

Machine-accessible metadata file describing the reported data: 10.6084/m9.figshare.12280112

## Background & Summary

The world’s biodiversity is impacted by increasing extinction rates^1,2^, particularly affecting freshwater aquatic ecosystems^3^. Water resource developments, invasive species, pollution, overharvesting and climate change are driving this decline^4,5^. Despite identification of long-term impacts, availability of biological data to track these changes remains poor, even though there is reasonably good data on the threats^6,7^, and effective means of tracking wetland loss using satellite image analyses^8^. In particular, there are relatively few large scale data sets available for mobile animals, extending over long periods of time, making it difficult to track long term effects of threats on freshwater biota.

Waterbird communities are a useful indicator group of organisms for monitoring changes to freshwater ecosystems^9^, given they are obligate aquatic organisms responsive to natural and anthropogenic changes in wetland ecosystems^10,11^. They are often considered a ‘flagship’ group for tracking changes in freshwater ecosystems and are included as a criterion for nomination of wetlands of international importance under the Ramsar Convention and the Important Bird and Biodiversity Areas designated by BirdLife International. They are also the centrepiece of migratory bird agreements (e.g. China Australia Migratory Bird Agreement). For further assessment of ecosystems, individual waterbird species may be categorised into functional response groups, reflecting different combinations of diet and habitat use^12,13^. This allows broad assessment of changes to wetland habitats^12^, including detrimental changes in ecosystem condition^14,15^.

Aerial surveys of waterbirds provide a rapid and cost effective method for collecting data over large spatial and long temporal scales^16–21^. Waterbirds are counted from a low flying aircraft (<50 m), with observers identifying and estimating the numbers of each species of waterbird on a wetland. The aircraft can manoeuvre and access large areas of wetland which may be inaccessible for ground or boat counts^20^. Given that aircraft can access remote areas at large spatial scales, there are also opportunities for spatial comparisons of data. Long term data can form the basis for analyses of changes in freshwater ecosystem condition.

**Fig. 1:**
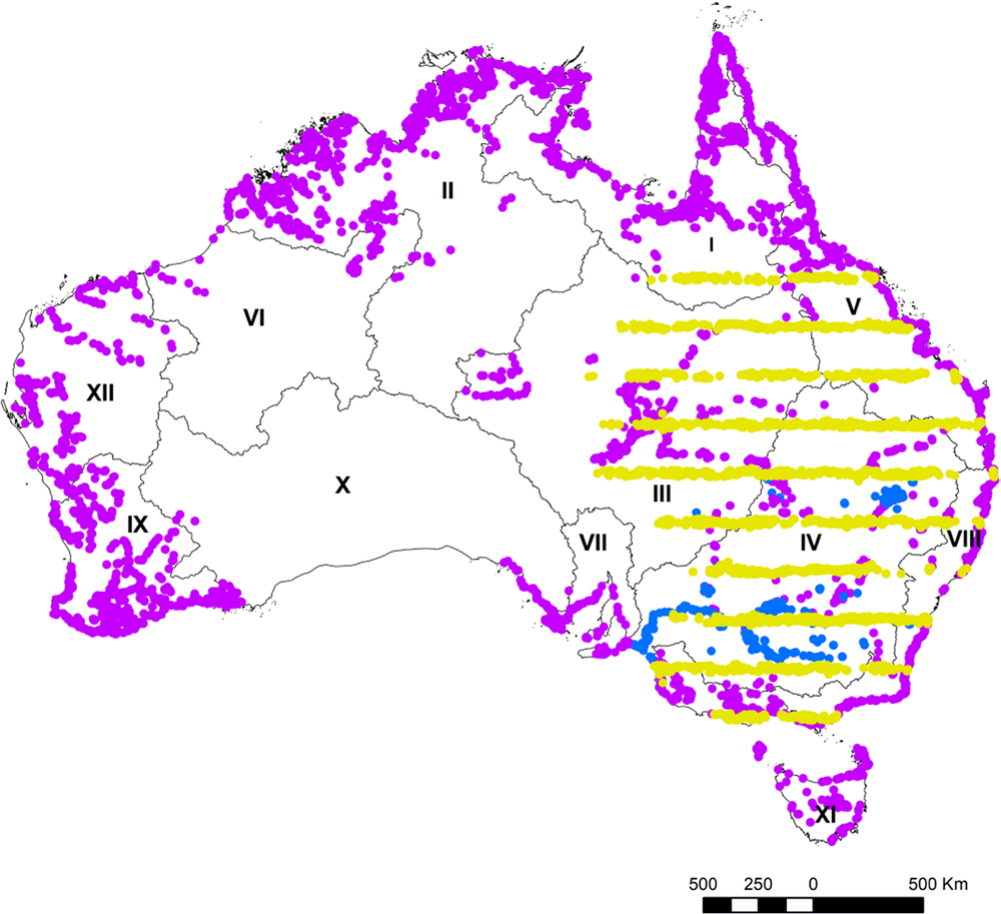
Aerial surveys showing wetlands (each dot) surveyed in the three waterbird aerial survey programs, across Australia, divided into the 12 major river basins (I-XII): Eastern Australian Waterbird Surveys (yellow circles, 10 survey bands, 30 km wide, 200 km separated, east to west); national waterbird survey (purple circles) and waterbird surveys of the major wetlands in the Murray-Darling Basin (IV) (blue circles). Major river basins numbered I) Carpentaria Coast, II) Tanami-Timor Sea Coast, III) Lake Eyre Basin, IV) Murray-Darling Basin, V) North East Coast, VI) Pilbara-Gascoyne VII) South Australian Gulf, VIII) South East Coast, IX) South West Coast, X) South Western Plateau, XI) Tasmania, XII) North Western Plateau.

The collection of this monitoring data on waterbirds in Australia is serving some key goals. First, the data are used each year by the governments of Victoria and South Australia to assess whether there are sufficient ‘game’ species (7 species^11^) for the setting of recreational duck hunting regulations. Game management authorities also assess the availability of wetland areas and non-target species, using data from aerial surveys^21^. These data are also critical for informing on the status and condition of rivers and important wetland areas for management. For example, the Murray-Darling Basin dataset, covering about one sixth of the continent in south-eastern Australia (Fig. 1), is tracking changes in waterbird abundances as a measure of river and wetland health. Australia has also used these data to track long-term changes reported in State of Environment Reporting at state and national levels. These data can also track long term changes in the ecological health of Ramsar-listed wetlands (e.g. Macquarie Marshes^22^). National legislation related to internationally significant Ramsar wetlands also necessitates assessment of potential changes to ecological character by potential developments. There is also a broad goal to track long term changes in species’ abundances and range for assessment of their Red-listing status. Finally, data for particular wetlands can be used to identify areas of high biodiversity value which can be prioritised for protection in the protected area network or other mechanisms.

We developed the Australian Waterbird Surveys (AWS) database to store and provide access to temporal and spatial waterbird data on individual species, their breeding status and estimates of wetland area, collected during annual aerial surveys, extending back to 1983. The data have been used for a range of published papers^11,12,14,20,21,23–43^. The core methodology for these surveys has remained the same. The database includes three principal survey programs: the Eastern Australian Waterbird Survey, the National Waterbird Survey and surveys of the major wetland sites in the Murray-Darling Basin (Fig. 1). Since 1983, the Eastern Australian Waterbird Survey has covered about a third of the continent each October, representing one of the larger and longer running biodiversity surveys in Australia, sampling wetland and waterbird communities across 2.7 million km^2^ of eastern Australia. In 2008, a relatively dry year, we did aerial surveys over most large wetlands across Australia during a period of two months comprising the National Waterbird Survey (Fig. 1). And since 2010, we have comprehensively surveyed all the major wetlands in the Murray-Darling Basin (Fig. 1).

## Methods

### Design

The Eastern Australian Waterbird Survey consists of 10 survey bands (30 km wide), extending from coast of eastern Australia through to the Northern Territory border (Fig. 1). Within each survey band, all rivers and wetlands (>1 ha) are marked and surveyed each October (1983–2019), currently providing more than three decades of data on up to 2,000 wetlands. In addition, small wetlands (<1 ha, e.g. small farm dams), not marked on 1:250,000 maps, are also surveyed in *ad-hoc* fashion while traversing between mapped units. These were surveyed reasonably consistently each year, within the survey bands, and their encounter rate was primarily dependent on their frequency of occurrence and whether other large wetlands were counted nearby. The number of wetlands surveyed each year varies with flooding and drying events. For the national survey in 2008 (30^th^ Sept–30^th^ November), we focused on all wetlands known to be significant for waterbirds, given that waterbirds concentrate on a relatively few large or productive wetlands^11,21^. This included all inland or coastal wetlands listed under the International Convention for Wetlands (Ramsar) and all sites listed in Directory of Important Wetlands in Australia (http://www.environment.gov.au/water/wetlands/australian-wetlands-database/directory-important-wetlands) (Fig. 1). To improve the efficiency of the aerial survey, satellite imagery (Landsat or MODIS) was also inspected prior to the survey to identify inundated or dry wetlands, allowing us to conserve resources and not survey dry wetlands, usually on the western extremities of the survey bands. Wetland spatial data were extracted from the 1:250,000 national waterbody layer^44^ and loaded on GPS systems. For the major wetlands in the Murray-Darling Basin, we surveyed all the major wetlands (2010–2019, including most Ramsar-listed sites), and all major floodplains and lakes (Fig. 1).

### Data collection

Waterbirds (including nests and broods) were counted from high-winged aircraft (e.g. Cessna 206 or 208) at 167–204 km hr^−1^ and a height of 30–46 m, within 150 m of the wetland’s shoreline where waterbirds concentrated^12^ (Tables 1 and 2). A front-right observer (navigator) and a back-left observer independently record counts on audio recorders, with their combined counts making up a completed count^21^. Counts are attributed on the recorder to a unique number for each wetland, and a geolocation (longitude, latitude), as well as the exact time of day the survey commenced. All timing is synchronised to GPS time – this enables audio counts to be linked to location via a GPS track log of the flight path (Table S1). Also, the percent fullness (inundated area) of each wetland is estimated, relative to the mapped high water mark^44^; inundated areas (ha) are also estimated for wetlands which are not mapped (Table 1). All waterbirds are identified to species, except those few that cannot be consistently identified to species’ level from the air and were grouped: small grebes (Australasian little grebe, hoary headed grebe), small egrets (Cattle egret, Little egret and Intermediate egret), terns and small and large migratory wading birds (Charadriformes) (Tables 1 and S2). Counts of no birds are also recorded, as are dry wetlands. Waterbirds are counted singly and in groups, progressively increasing up to 1,000 individuals.

**Table 1:** Data collected during each aerial survey of waterbirds on a wetland, comprising the data record.

Descriptor	Record	Protocol	Data
Waterbird species	Identification and count of each species on a particular wetland	Counts are recorded into tape recorders for a front right observer and a left back observer. This is then totalled for the wetland.	Estimate of total of each species and the number of nests and broods (numeric)
Wetland	Name of wetland if available, otherwise a location provided	The name of the wetland is read from a map. For the Eastern Australian Waterbird survey, the location of the survey band is also included. For wetlands not named, a latitude and longitude are read from the GPS and recorded. For all wetlands, an assessment is made of the percentage fullness of the wetland and, for wetlands not named, the area is estimated.	Name of the wetland (text), percentage full relative to high water mark (numeric) and for unnamed wetlands the area in hectares (numeric)
Type of count	One of three types of count is recorded: total count, proportion count and transect count	A decision is made by the navigator in relation to the type of count, depending on the concentration of waterbirds and the complexity of the wetland. For proportion counts, the navigator (right front observer) estimates the proportion of the wetland counted	Total (1), Transect (2), Proportion (3) (numeric)
Replicate	More than one count may be done at a wetland at a particular time, representing an immediate replicate	The first count of a wetland may be followed immediately by a second count of the wetland. This approach is utilised in the aerial survey of major wetlands in the Murray-Darling Basin, using two replicates. Where only one count is done, the replicate recorded is 1.	Replicates (1, 2…) (numeric)

**Table 2:** Main descriptors and descriptions of stored invariant data in the Australian Waterbird Survey database and their links (see Table S1 for detailed data dictionary).

Descriptor	Description	Links
Waterbird species	Lists all waterbird species identified and counted on aerial surveys of waterbirds. Some species are grouped (small grebes, egrets, terns, large waders, small waders).	This list is linked to the Australian Faunal Directory (AFD) https://biodiversity.org.au/afd/home with each species assigned an AFD code which is regularly updated. Each waterbird species is also assigned a functional response group category in one of the following piscivore, herbivore, ducks and grebes, large wading birds and shorebirds (Table S2).
Wetland	Names are taken from the 1:250,000 waterbody map layer, with areas calculated using GIS polygons. Wetlands (>1 ha) without names are given a name related to the nearest feature on the map and its direction. Small wetlands are given a latitude and longitude during each survey.	Wetlands are linked to large river basins and also, when within a large floodplain complex, they can have a group system name (e.g. Lowbidgee wetlands). This allows for hierarchical searching and reporting.
Observer	Each observer has a number which is tagged to their observations.	Contact details are recorded for all observers.
Survey program	There are three major survey programs: Eastern Australian Aerial Survey, National Aerial Survey and Survey of major wetlands in the Murray-Darling Basin. Other survey programs can also be added.	Survey programs are primarily used for separate reporting to authorities with different jurisdictions.

Three counting techniques are used: total counts, proportion counts and transect counts. For total counts, all birds are counted during a circumnavigation of the wetland, the preferred method for wetlands with large concentrations of waterbirds. For proportion counts, a proportion (usually >50%) of a large wetland with few waterbirds (e.g. large dam) is surveyed, with counts extrapolated to give total counts. For the final transect method, waterbirds are counted within 200 m-wide transects (100 m on each side of the aircraft, delineated by tape on each aircraft wing strut), a technique only used for braided complex large wetlands. Total estimates are formed from the relative area of the transects, compared to total area flooded.

### Data entry, processing and output

Following recording of a day’s survey, audio data are transcribed onto data sheets and then entered into a computer data file through a developed JSON (JavaScript Object Notation) application (Table S1), loaded with the name of the survey program, all wetland locations, names, waterbird species’ names and dates. This reduces potential transcription errors in relation to data entry. During data entry, a time-stamped GPS track of the survey path (Table S1) is used to ensure that any navigational issues, such as erroneous georeferencing, are corrected using the GPS time recorded during the surveys. Wetlands not appearing on 1:250,000 map layers^44^ are given a geolocation, estimated percent full and inundated area that is recorded during the survey. Wetland geolocation and area for mapped and numbered wetlands is obtained from the waterbody map layer. Each day of the survey is entered separately for each observer. All data are incorporated into a MySQL database, allowing subsequent error checking, identification and correction.

As each record of data is entered, it is checked by JSON for errors by checking non bird count data is consistent among observers; for example, date and time of survey, unit name, percent full and area must match. Second, for mapped wetland units, their numbers, geolocations and areas are checked against that obtained from the waterbody map layer. Ultimately, a count of the numbers of each species (and any nests or broods) are totalled for each wetland for total counts and extrapolations of proportions or transect counts (Tables 1 and S1). This defines the information for the waterbird count in the data record. The data record for the wetland can then displayed in a Google Maps interface, supported by the Australian Waterbird Surveys database (https://aws.ecosystem.unsw.edu.au/). Australian Waterbird Surveys is a relational database management system, originally constructed in MSAccess using structured query language (SQL) to link with GIS software and deliver the database online via a server. It allows flexible retrieval of the temporal and spatial dimensions of the data.

## Data Records

A data record consists of counts of each waterbird species (abundance, number of nests and broods) for each wetland, during a particular survey (Tables 1 and S1). In addition, the type of count and replicate number of the count are included (Table 1). This data record is linked to data for waterbird species (Table S2), the wetland (linked to wetland complex and river basin), observer and survey program (Table 2). The survey program defines the spatial and temporal scale of the aerial survey of waterbirds which can depend on the survey design and level of funding. The most comprehensive dataset in the Australian Waterbird Survey database, the Eastern Australian Waterbird Survey dataset spans 37 years (1983–2019) over up to 2,000 wetlands and ∼100 waterbird species (Fig. 1, Table S2). The National Waterbird Survey in 2008, involved a survey of 4,858 wetlands across Australia between the end of September and end of November, Fig. 1). The third survey program focuses on the major wetlands in the Murray-Darling Basin Surveys, surveyed each year in October-November since 2010, with wetlands along the River Murray surveyed since 2007 (Fig. 1).

Like all relational database systems, data records are supported by lookup tables of invariant data (Table 1). The Wetlands table and related tables contains data relating to wetland physical and cadastral attributes (name, geographic coordinates, mapped area, unique wetland Identification number, river basin, drainage division and state). The table relating to different waterbird species is linked to the Australian Faunal Directory (Tables 2 & S2) and the database also holds records of the different observers used on aerial surveys (Table 2). All these aerial survey data, including available data entry files and tracklogs can be accessed at figshare^45^.

## Technical Validation

All observers were trained to rapidly count and identify the different waterbird species, including recognition of their wing patterns from above. They were also trained, sitting behind an experienced observer linked by an intercom which allowed the trainee to hear identifications and counts of waterbirds. Wildlife counting software was also employed to improve and evaluate rapid counting skills (http://www.fws.gov/waterfowlsurveys/). All observers were trained over a period of at least 50 hours and their data evaluated by comparison to an experienced observer.

Large shallow or complex braided wetlands sometimes had waterbirds distributed across their surface. These required either transect counts or total counts of different parts of the wetland. For wetlands where only proportion counts were used, surveys generally cover more than half the area counted with some areas omitted where manoeuvrability of the aircraft is limiting (e.g. long convoluted bays). Wetlands where only about half the wetland are counted, were usually large dams with relatively few waterbirds. Total counts were usually attempted when large numbers of waterbirds were encountered.

Aerial surveys of multispecies flocks of waterbirds are inherently imprecise but can separate orders of magnitude variation reasonably well^20^. Spatial and temporal changes in waterbird populations often considerably exceed the measurement variation experienced with aerial surveys of waterbirds^11,14^. For major wetlands in the National Wetland and Murray-Darling Basin survey programs, replicate counts allow for some measurement of this variation.

Wetland locations and boundaries are difficult to map accurately, particularly in areas of low topographic relief and unpredictable flooding patterns, experienced across large parts of the Australian continent. Geographic details for small (<1 ha) wetlands are reliant on georeferencing from the aircraft which may be uncertain depending on the proximity of the aircraft when the reading is provided, compared to the actual location of the wetland. Similarly, some linear features such as streams and small watercourses are mapped as polylines and hence areas are not available; assessment of wetland area at the time of the survey were used to provide areas for these freshwater systems.

## Usage Notes

The graphical user interface on the Australian Waterbird Survey database allows access to data through a spatial portal. Users can access and download data by specifying an area by defining a circle or a box over a selected region on the Google Maps interface. This allows selection of different aerial survey programs and downloading of data as text (csv) file.

## Supplementary information

Supplementary Information
