# Supplementary material for: Aerial surveys of waterbirds in Australia
Source: Sci Data. 2020 Jun 10;7:172. doi: 10.1038/s41597-020-0512-9 (PMC7287086; doi:10.1038/s41597-020-0512-9)
Supplement: Supplementary file 1 — Supplementary Information [file 41597_2020_512_MOESM1_ESM.docx]

### Supplementary information

**Contents**

[Supplementary Table 1. Data dictionary for fields in the dataset for Aerial Surveys of Waterbirds. Full data set is available^45^. 2](#_Toc38722224)

[Supplementary Table 2. Common names of waterbird species, their functional groups (La–large wader, Pi – piscivore, Sh – shorebird, Du – duck, He – herbivore), species’ codes and ordered by CAVS (Census of Australian Vertebrate Species) number (NA-not applicable) in the Australian Faunal Directory 4](#_Toc38722225)

# Supplementary Table 1. Data dictionary for fields in the dataset for Aerial Surveys of Waterbirds. Full data set is available^45^.

| **Data collection** | **Data fields** | **Data description** | **Data type** |
| --- | --- | --- | --- |
| Australian Waterbird Survey |  |  |  |
|  | Program | Survey Program Name: Eastern Australian Waterbird Survey (EAWS), National Waterbird Survey, or Murray Darling Basin (MDB) Survey | text |
|  | Year | Year of survey YYYY | text |
|  | Wetland ID | Unique identifier applied to each surveyed wetland. Wetland ID corresponds to the EAWS and National Survey programs, while Identifier relates to the MDB Surveys | text |
|  | Band | Relates to survey band, EAWS only. | numeric |
|  | Number | Unique wetland number within the survey band | numeric |
|  | Subnumber | Unique number assigned to each defined wetland subunit. | numeric |
|  | Name | Wetland name | text |
|  | Common_name | Common name of species. ‘Zero count’ represents no birds present. | text |
|  | Scientific_name | Genus followed by species name (for nil counts this field is blank) | text |
|  | Functional_group | Functional group of waterbird species reflecting broad feeding and foraging preference | text |
|  | Species_code | Three letter species code. NIL is used for a zero count (no birds present) | text |
|  | Replicate | Count replicate 1, 2 or more. For some wetlands multiple counts (replicates) are undertaken. | text |
|  | Sum_of_Count | Number of birds of corresponding species counted | numeric |
|  | Sum_of_Nests | Number of nests of corresponding species counted | numeric |
|  | Sum_of_Broods | Number of broods of corresponding species counted | numeric |
|  | Wetland_Area | Total wetland area in hectares - derived from 250K waterbody layer where possible. Field may be blank for polygons that include multiple subunits (subwetlands) | numeric |
|  | Percent_full | Proportion of the wetland (surface area) inundated by water at the time of survey. Field may be blank for polygons that include multiple subunits (subwetlands) | numeric |
|  | Survey_Wetland_Area | Estimated wetland area (ha) at time of survey. Field may be blank for polygons that include multiple subunits (subwetlands) | numeric |
|  | LatitudeDec | Latitude of polygon centroid in decimal degrees (dd.mm) | numeric |
|  | LongitudeDec | Longitude of polygon centroid in decimal degrees (dd.mm) | numeric |
|  | Spatial_accuracy | Accuracy of location. 1 <1 minute Latitude/Longitude, determined from survey (Aviation certified GPS) ; 2 <6 minutes Latitude/Longitude, no gps available; determined from combination of map, time and nearest known locations; 3 < 1 degree Latitude/Longitude, no gps available; determined from combination of map, time and nearest known locations | numeric |
| Australian Aerial Waterbird Survey Tracklogs |  | Aviation GPS flight path track log; includes travel to and from count sites. Positioning is logged every 1-10 seconds, except sometimes longer in 2008; not all data fields available in all tracklog files; tracklogs for some years (e.g. 2012) incomplete due to equipment failure. |  |
|  | Index | Sequential count of track point | numeric |
|  | Time\Date | Time and date stamp | numeric |
|  | Elevation | Height above ground level | numeric |
|  | Leg length | Length span of leg (a leg comprises two adjacent track points, current and next in the sequence) | numeric |
|  | Leg time | Time span of leg | numeric |
|  | Leg speed | Aircraft speed of leg; incorrect speeds may display in some fields | numeric |
|  | Leg course | Compass bearing of leg | numeric |
|  | Position | Latitude and longitude of track point in decimal degrees; Geodetic datum is WGS 84 | numeric |
| Australian Aerial Waterbird Survey JSON Data input files |  | Raw input count data comprising each observer’s count from one side of the aircraft only; forming partial counts to be added to the other observer’s count (other side of plane). These partial counts are combined within the survey database to provide a single estimate (a data record) for each wetland surveyed. These JSON files are only available from 2014 onwards. Most of the data fields are identical to those used in the data records. |  |
|  | Sheet | Data sheet number | numeric |
|  | Day | Day of survey program | numeric |
|  | Date | Date time stamp | numeric |
|  | SequenceNum | Sequential number of each observer’s count for their unique wetland for a given day | numeric |
|  | Name | Observer name | text |
|  | RecNo | Observer number | numeric |
|  | Time | Time of day – synchronised with GPS clock | numeric |
|  | Wetland code | Wetland index - includes survey band, wetland number and subnumber | Alpha-numeric |
|  | GPS | Latitude and longitude coordinates of aircraft at time of count in decimal degrees (Geodetic datum is WGS 84) |  |
|  | Proportion | Estimated proportion of wetland counted | numeric |
|  | Filled | As for Percent_full above | numeric |
|  | Count_type | Count method (total=1, transect=2, proportional=3) | text |
|  | Type | Wetland type (natural=1, artificial=2) | text |
|  | Bird | As for Species_code above | text |
|  | Entries | As for Sum_of_Count above | numeric |
|  | Current_area | As for Survey_Wetland_Area above | numeric |

Supplementary Table 2. Common names of waterbird species, their functional groups (La–large wader, Pi – piscivore, Sh – shorebird, Du – duck, He – herbivore), species’ codes and ordered by CAVS (Census of Australian Vertebrate Species) number (NA-not applicable) in the Australian Faunal Directory <https://biodiversity.org.au/afd/home> **surveyed during aerial surveys.**

| **Common Name** | **Scientific Name** | **Functional**  **group** | **Species’ code** | **CAVS** |
| --- | --- | --- | --- | --- |
| Black-tailed Native-hen | *Tribonyx ventralis* | He | BTN | 0055 |
| Dusky Moorhen | *Gallinula tenebrosa* | He | MHE | 0056 |
| Purple Swamphen | *Porphyrio porphyrio* | He | SHE | 0058 |
| Eurasian Coot | *Fulica atra* | He | COT | 0059 |
| Great Crested Grebe | *Podiceps cristatus* | Pi | GCG | 0060 |
| Australasian Grebe | *Tachybaptus novaehollandiae* | Du | ALG | 0061 |
| Hoary-headed Grebe | *Poliocephalus poliocephalus* | Du | HHG | 0062 |
| Small grebes  Hoary-headed Grebe  Australasian Grebe | Small grebe species  *Poliocephalus poliocephalus*  *Tachybaptus novaehollandiae* | Du | GRE | T180 |
| Great Cormorant | *Phalacrocorax carbo* | Pi | GRC | 0096 |
| Little Black Cormorant | *Phalacrocorax sulcirostris* | Pi | LBC | 0097 |
| Pied Cormorant | *Phalacrocorax varius* | Pi | PCO | 0099 |
| Little Pied Cormorant | *Phalacrocorax melanoleucos* | Pi | LPC | 0100 |
| Darter | *Anhinga melanogaster* | Pi | DAR | 0101 |
| Australian Pelican | *Pelecanus conspicillatus* | Pi | PEL | 0106 |
| Whiskered Tern | *Chlidonias hybridus* | Pi | MST | 0110 |
| Caspian Tern | *Sterna caspia* | Pi | CST | 0112 |
| Gull-billed Tern | *Sterna nilotica* | Pi | GBT | 0111 |
| Silver Gull | *Larus novaehollandiae* | Pi | SGU | 0125 |
| Pacific Gull | *Larus pacificus* | Pi | PGU | 0126 |
| Terns undifferentiated  Whiskered Tern  White-winged Black Tern  Lesser Crested Tern  Crested Tern  Common Tern | Tern species- includes:  *Chlidonias hybridus*  *Chlidonias leucopterus*  *Sterna bengalensis*  *Sterna bergii*  *Sterna hirundo* | Sh | TNS | NA |
| Pied Oystercatcher | *Haematopus longirostris* | La | POC | 0130 |
| Sooty Oystercatcher | *Haematopus fuliginosus* | La | SOC | 0131 |
| Masked Lapwing | *Vanellus miles* | Sh | MLW | 0133 |
| Banded Lapwing | *Vanellus tricolor* | Sh | BDP | 0135 |
| Black-winged Stilt | *Himantopus himantopus* | Sh | WHS | 0146 |
| Banded Stilt | *Cladorhynchus leucocephalus* | Sh | BST | 0147 |
| Red-necked Avocet | *Recurvirostra novaehollandiae* | Sh | AVO | 0148 |
| Large Waders  Bar-tailed Godwit  Black-tailed Godwit  Eastern Curlew  Little Curlew  Whimbrel | Large wader species - includes:  *Limosa lapponica*  *Limosa limosa*  *Numenius madagascariensis*  *Numenius minutus*  *Numenius phaeopus* | Sh | LGW | T181 |
| Small Waders  Grey Plover  Pacific Golden Plover  Mongolian Plover  Double-banded Plover  Black-fronted Plover  Red-capped Plover  Ruddy Turnstone  Grey-tailed Tattler  Common Sandpiper  Marsh Sandpiper  Terek Sandpiper  Greenshank  Red Knot  Great Knot  Sharp-tailed Sandpiper  Red-necked Stint  Curlew Sandpiper  Broad-billed Sandpiper  Red-kneed Dotterel  Latham's snipe | Small wader species - includes:  *Pluvialis squatorola*  *Pluvialis fulva*  *Charadrius mongolus*  *Charadrius bicinctus*  *Charadrius melanops*  *Charadrius ruficapillus*  *Arenaria interpres*  *Tringa brevipes*  *Tringa hypoleucos*  *Tringa stagnatilis*  *Tringa terek*  *Tringa nebularia*  *Calidris canutis*  *Calidris tenuirostris*  *Calidris acuminata*  *Calidris ruficollis*  *Calidris ferruginea*  *Limicola falcinellus*  *Erthrogonys cintus*  *Gallinago hardwickii* | Sh | SMW | T181 |
| Comb-crested Jacana | *Irediparra gallinacea* | Du | JAC | 0171 |
| Brolgas  Brolga  Sarus Crane | Brolga species- includes:  *Grus rubicunda*  *Grus antigone* | La | BRL | 0177 |
| Glossy Ibis | *Plegadis falcinellus* | La | GLI | 0178 |
| Australian White Ibis | *Threskiornis molucca* | La | WHI | 0179 |
| Straw-necked Ibis | *Threskiornis spinicollis* | La | SNI | 0180 |
| Royal Spoonbill | *Platalea regia* | La | RSB | 0181 |
| Yellow-billed Spoonbill | *Platalea flavipes* | La | YSB | 0182 |
| Black-necked Stork | *Ephippiorhynchus asiaticus* | La | JAB | 0183 |
| Great-billed Heron | *Ardea sumatrana* | La | GBH | 0184 |
| Great Egret | *Ardea alba* | La | LGE | 0187 |
| Unidentified Egret  Little Egret  Cattle Egret  Intermediate Egret | Egret species  *Egretta garzetta*  *Ardea ibis*  *Ardea intermedia* | La | EGR | T179 |
| White-faced Heron | *Egretta novaehollandiae* | La | WFH | 0188 |
| Pacific Heron | *Ardea pacifica* | La | WNH | 0189 |
| Pied Heron | *Ardea picata* | La | PIH | 0190 |
| Eastern Reef Egret | *Egretta sacra* | La | ERE | 0191 |
| Rufous Night Heron | *Nycticorax caledonicus* | La | NKE | 0192 |
| Striated Heron | *Butorides striatus* | La | STH | 0193 |
| Cape Barren Goose | *Cereopsis novaehollandiae* | He | CBG | 0198 |
| Magpie Goose | *Anseranas semipalmata* | He | MPG | 0199 |
| Cotton Pygmy-Goose | *Nettapus coromandelianus* | Du | WPG | 0200 |
| Green Pygmy-Goose | *Nettapus pulchellus* | Du | GPG | 0201 |
| Australian Wood Duck | *Chenonetta jubata* | He | WDU | 0202 |
| Black Swan | *Cygnus atratus* | He | BSW | 0203 |
| Wandering Whistling-Duck | *Dendrocygna arcuata* | Du | WWD | 0204 |
| Plumed Whistling-Duck | *Dendrocygna eytoni* | He | GWD | 0205 |
| Radjah Shelduck | *Tadorna radjah* | Du | BKU | 0206 |
| Australian Shelduck | *Tadorna tadornoides* | He | MNU | 0207 |
| Pacific Black Duck | *Anas superciliosa* | Du | BDU | 0208 |
| Chestnut Teal | *Anas castanea* | Du | CTL | 0210 |
| Grey Teal | *Anas gracilis* | Du | GTL | 0211 |
| Australasian Shoveler | *Anas rhynchotis* | Du | BWS | 0212 |
| Pink-eared Duck | *Malacorhynchus membranaceus* | Du | PED | 0213 |
| Freckled Duck | *Stictonetta naevosa* | Du | FDU | 0214 |
| Hardhead | *Aythya australis* | Du | HHD | 0215 |
| Blue-billed Duck | *Oxyura australis* | Du | BBU | 0216 |
| Musk Duck | *Biziura lobata* | Du | MDU | 0217 |
| Domestic Goose | *Anser* sp*.* | He | DMG | T183 |
| Kelp Gull | *Larus dominicanus* | Pi | KGU | 0981 |
